# Spectral Signatures of Oxidation States in a Manganese‐Oxo Cubane Water Oxidation Catalyst

**DOI:** 10.1002/chem.202102583

**Published:** 2021-10-15

**Authors:** Sebastian Mai, Sarah Klingler, Ivan Trentin, Julian Kund, Marcus Holzer, Anastasia Andreeva, Robert Stach, Christine Kranz, Carsten Streb, Boris Mizaikoff, Leticia González

**Affiliations:** ^1^ Institute of Theoretical Chemistry Faculty of Chemistry University of Vienna Währinger Straße 17 1090 Vienna Austria; ^2^ Institute of Analytical and Bioanalytical Chemistry Ulm University Albert-Einstein-Allee 11 89081 Ulm Germany; ^3^ Institute of Inorganic Chemistry I Ulm University Albert-Einstein-Allee 11 89081 Ulm Germany; ^4^ Hahn-Schickard Sedanstraße 14 89077 Ulm Germany

**Keywords:** Density functional calculations, IR spectroscopy, Polyoxometalates, UV/Vis spectroscopy, Water splitting

## Abstract

We report IR and UV/Vis spectroscopic signatures that allow discriminating between the oxidation states of the manganese‐based water oxidation catalyst [(Mn_4_O_4_)(V_4_O_13_)(OAc)_3_]^3−^. Simulated IR spectra show that V=O stretching vibrations in the 900–1000 cm^−1^ region shift consistently by about 20 cm^−1^ per oxidation equivalent. Multiple bands in the 1450–1550 cm^−1^ region also change systematically upon oxidation/reduction. The computed UV/Vis spectra predict that the spectral range above 350 nm is characteristic of the managanese‐oxo cubane oxidation state, whereas transitions at higher energy are due to the vanadate ligand. The presence of absorption signals above 680 nm is indicative of the presence of Mn^III^ atoms. Spectroelectrochemical measurements of the oxidation from [Mn${}_{2}^{\mathrm{III}}$ Mn${}_{2}^{\mathrm{IV}}$ ] to [Mn${}_{4}^{\mathrm{IV}}$ ] showed that the change in oxidation state can indeed be tracked by both IR and UV/Vis spectroscopy.

## Graphical abstract

**Tracing fingerprints**. Key IR and UV/Vis spectroscopic signatures to differentiate oxidation states of the manganese‐oxo vanadate catalyst [(Mn_4_O_4_)(V_4_O_13_)(OAc)_3_]^*n*–^ are identified. During oxidation from [Mn${}_{2}^{\mathrm{III}}$ Mn${}_{2}^{\mathrm{IV}}$ ] to [Mn${}_{4}^{\mathrm{IV}}$ ], in the UV/Vis spectrum a band at about 570 nm increases while another one at 850 nm vanishes. In the IR spectra, oxidation leads to a remarkable shift of the V=O stretch mode to higher energies.

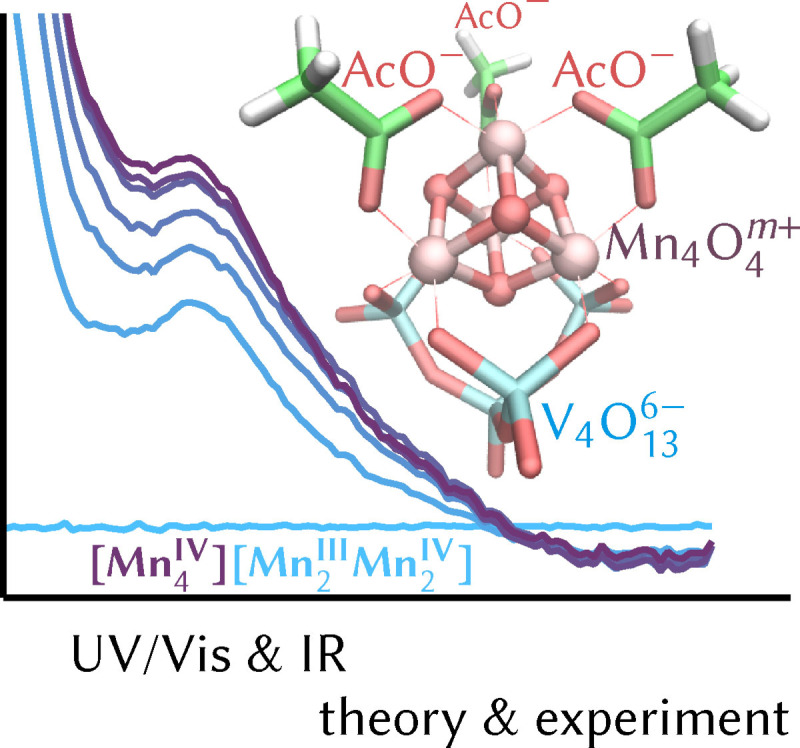

## Introduction

**Figure 1:**
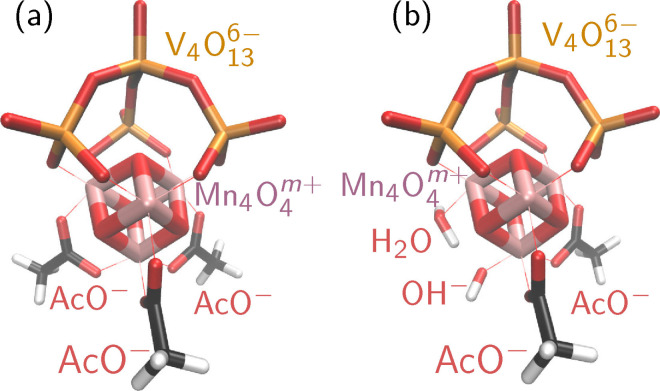
Three‐dimensional depictions of the MnV WOC precatalyst with three acetate ligands (a) and the catalytically active form (b), where one of the acetates is exchanged for a water ligand and a hydroxide ligand.

Artificial photosynthesis^[1]^ is a promising avenue for accessing carbon‐neutral energy supplies. One way to realize it is photocatalytic water splitting, where the solar energy is used to produce molecular hydrogen (2H_2_O→O_2_+2H_2_). Water splitting requires highly specialized catalysts to be efficient, what spurred intensive research since decades. In particular, water oxidation catalysis (2H_2_O→O_2_+4H^+^+4e^–^) is difficult to realize, as catalysts need to be oxidatively robust, support multiple oxidation states to store the required four oxidation equivalents, enabling proton‐coupled electron transfer, and have several other requirements.[^2^, ^3^, ^4^] A highly active water oxidation catalyst (WOC) is the oxygen‐evolving complex within photosystem II, which contains an Mn_4_CaO_5_ cluster as the reactive center. Inspired by it, other artificial Mn‐based catalysts were developed with an Mn_4_O_4_ cubane core.[^5^, ^6^, ^7^, ^8^, ^9^] The polyoxometalate‐stabilized compound^[8]^ [(Mn_4_O_4_)(V_4_O_13_)(OAc)_3_]^3−^ (labelled “MnV WOC”, Figure 1a) was recently used to catalyze light‐driven water oxidation in a water‐acetonitrile mixture, achieving a turn‐over frequency of 3.6 s^−1^ and a turn‐over number of approximately 12,000.^[10]^

In order to optimally utilize the catalytic activity of this MnV WOC – and to eventually design more effective catalysts – it is mandatory to fully understand the microscopic mechanistic details of the catalytic reaction. Previous computational, electrochemical, and spectroscopic work^[11]^ has shown that the activation of the Mn WOC involves the exchange of an acetate for a water ligand and a hydroxide ligand (Figure 1b), accompanied by two oxidation steps that bring the compound from the initial [Mn${}_{2}^{\mathrm{III}}$ Mn${}_{2}^{\mathrm{IV}}$ ] state to [Mn${}_{4}^{\mathrm{IV}}$ ]. Based on what is known about the catalytic cycle of the natural oxygen‐evolving complex,[^12^, ^13^, ^14^] the actual catalytic cycle of MnV‐WOC will then also involve multiple oxidation states and different ligand transformations not yet fully identified.^[15]^ In order to enable the spectroscopic characterization of the catalytic mechanism, here we computationally predict the infrared and UV/Vis absorption spectroscopic signals of the different oxidation and ligand states of the WOC and verify those with in‐situ spectroelectrochemical (SEC) IR and UV/Vis measurements.

## Results and discussion

### Oxidation states and ligands

The pristine MnV WOC was identified to have a [Mn${}_{2}^{\mathrm{III}}$ Mn${}_{2}^{\mathrm{IV}}$ ] configuration, although [Mn^III^Mn${}_{3}^{\mathrm{IV}}$ ] and [Mn${}_{4}^{\mathrm{IV}}$ ] states are also accessible electrochemically.^[8]^ We hypothesize that the activated species also goes through [Mn${}_{3}^{\mathrm{III}}$ Mn^IV^] and possibly also [Mn${}_{4}^{\mathrm{III}}$ ] during water oxidation catalysis.^[11]^ Note that in order to start the water oxidation catalysis, accessing a (presumably short‐lived) oxidation state higher than [Mn${}_{4}^{\mathrm{IV}}$ ] is required based on the current understanding of the system.[^8^, ^11^] Thus, our goal is to enable the identification of these different oxidation states (Table 1) by means of spectroscopy.

**Table 1:** Considered oxidation states of the Mn WOC with different labels and total associated charges.

Oxidation state	Label${}^{\left[a\right]}$	Label${}^{\left[b\right]}$	Charge
[${Mn}_{4}^{IV}$ ]	Mn4444	*S*_3_	1−
[Mn^III^ ${Mn}_{3}^{IV}$ ]	Mn3444	*S*_2_	2−
[${Mn}_{2}^{III}{Mn}_{2}^{IV}$ ]	Mn3344	*S*_1_	3−
[${Mn}_{3}^{III}$ Mn^IV^]	Mn3334	*S*_0_	4−
[${Mn}_{4}^{III}$ ]	Mn3333	*S*_–1_	5−

$\left[a\right]$ These labels explicitly specify the oxidation state of each Mn atom. $\left[b\right]$ Nomenclature as in the Kok‐cycle in PS II.^[16]^

For each oxidation state, the MnV WOC exhibits multiple thermally accessible local minima because each Mn^III^ atom assumes an elongated octahedral coordination triggered by the Jahn–Teller effect of the *d* ^4^ electron configuration. Previous computational work[^11^, ^17^] showed that out of the 256 possible Jahn‐Teller axes arrangements, 52 are distinguishable within *C*_3*v*_ symmetry, and 12 of these are stable minima (1, 2, 3, 4, and 2 for oxidation states Mn4444 to Mn3333). In the present work, we primarily focus on the spectra of the most stable minimum of each oxidation state; however, we also investigate the spectral effects of the structural flexibility due to the other minima. For the same reason, we also compute spectra of all accessible minima of the activated complex (2, 6, 7, 4, 1 for oxidation states Mn4444 to Mn3333), where one of the acetates is dissociated and the two free coordination sites are occupied by a water and a hydroxide ion.^[11]^

### Computed infrared spectra

**Figure 2:**
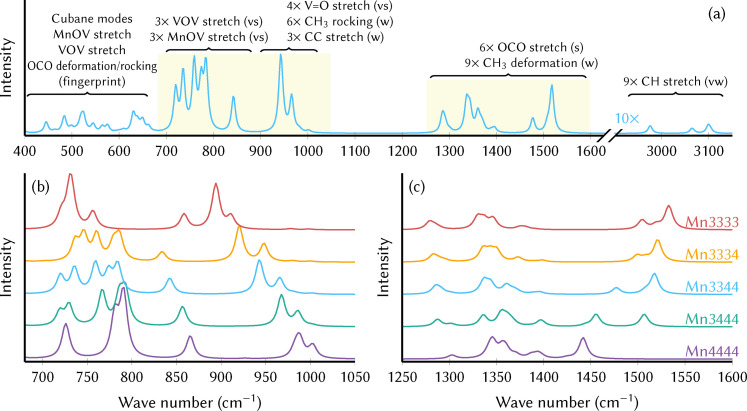
(a) Simulated IR spectrum of the Mn3344 oxidation state of the precatalyst and assignment of important vibrations. (b, c) Simulated IR spectra of different oxidation states in the spectral ranges of interest (yellow shading in (a)). Only the spectra of the lowest‐energy local minima are shown.

Density functional theory (DFT) was employed to compute the IR spectra of the thermodynamically most important minimum of each oxidation state of MnV‐WOC in solution. Figure 2a presents the simulated IR spectrum of the Mn3344 oxidation state of the precatalyst and the vibrational mode assignment; the assignment of a related experimental IR spectrum and a comparison of simulation and experiment are shown in the Supporting Information in Figures S1 and S2 (Section S1.1). We briefly discuss this spectrum as a prototype for the spectra of the other oxidation states, ligand configurations, and local minima. The spectrum is well structured with clearly separated regions. The CH stretch vibrations of the acetate ligands are located around 3000 cm^−1^, but are very weak. The 1250–1600 cm^−1^ region contains only vibrations localized on the acetate ligands, with six strong OCO stretch vibrations that lead to a variable number of bands depending on the oxidation state. A very prominent double peak can be seen in the 900–1020 cm^−1^ region, arising from the four V=O stretch vibrations. The same region also contains nine further acetate vibrations, but they are weak and concealed by the V=O stretch bands. In the 700–880 cm^−1^ region, six very intense bands are produced by three VOV asymmetric stretch modes and three MnOV asymmetric stretch modes. No other vibrations of any intensity are located in this region. Below 700 cm^−1^, additional bands of medium intensity are observed, although many weak transitions are also located in this fingerprint region.

In Figure 2b and 2c, we present the simulated IR spectra for different oxidation states in the spectral regions that are most promising for distinguishing these states. Around 700–800 cm^−1^, the spectrum evolves from only two bands in the *C*_3*v*_‐symmetric Mn4444 state to five distinct bands in the reduced species, where the different Jahn‐Teller axes of the Mn^III^ atoms lower the symmetry and induce band splitting. The isolated band around 850 cm^−1^ and in particular the double peak around 900–1000 cm^−1^ show clear and systematic red shifts when going to more reduced species. Furthermore, the bands in the 1450–1550 cm^−1^ region (Figure 2c) show an easily visible trend. It arises from the fact that an acetate bonded to Mn^IV^ atoms shows an asymmetric OCO stretch band around 1420–1470 cm^−1^, whereas one bonded to Mn^III^ experiences a blueshift to about 1500–1540 cm^−1^.

The most interesting features for the spectroscopic characterization of the oxidation state of the MnV WOC are certainly the two bands around 800–1000 cm^−1^. The computed positions of the involved transitions for the different oxidation states are given in Table 2. A possible explanation for the strong dependence of the V=O stretch frequencies on the oxidation state of the Mn_4_O_4_ cubane could be an inductive effect of the Mn_4_O_4_ cubane. A more positively charged cubane (at high oxidation states) induces a slight reduction of electron density at the V atoms, which thus become more Lewis‐acidic and form a stronger bond to the O atoms. The stronger bond consequently leads to higher vibrational frequencies for higher oxidation states.

**Table 2:** Shift of V=O and VOV frequencies (cm^−1^) with oxidation state.

Ox. state	$\nu_{1}^{\left[a\right]}$	$\nu_{2}^{\left[a\right]}$	$\nu_{3}^{\left[a\right]}$	$\nu_{4}^{\left[a\right]}$	$\nu_{5}^{\left[b\right]}$
Mn4444	1003	989	986	982	865
Mn3444	986	968	967	967	857
Mn3344	967	947	943	942	842
Mn3334	948	926	921	920	834
Mn3333	910	895	893	891	858

$\left[a\right]$ : *ν*_1–4_ combinations of V=O stretches. $\left[b\right]$ : *ν*_5_ symmetric combination of asymmetric VOV stretches.

As mentioned above, each oxidation state possesses several thermally accessible local minima due to the different Jahn‐Teller configurations, each of which might produce slightly different IR spectra. Moreover, the exchange of an acetate ligand for H_2_O and OH^−^ during catalyst activation can also affect the spectra. As shown in Figure 2, the marker bands in the 850–1000 cm^−1^ and 1450–1550 cm^−1^ ranges are virtually unaffected by these structural changes (i. e., within each color). On the contrary, the oxidation state (different colors) significantly modulates the spectra. This is a strong indication that, as long as the oxidation state does not change, the position of the marker bands will not be affected during the precatalyst activation, catalytic cycle, or catalyst regeneration. In other words, the marker bands can serve to unambiguously identify the oxidation states in the reaction mixture, at least if the marker bands are not concealed by absorption of other constituents.

### Experimental infrared spectra

**Figure 3:**
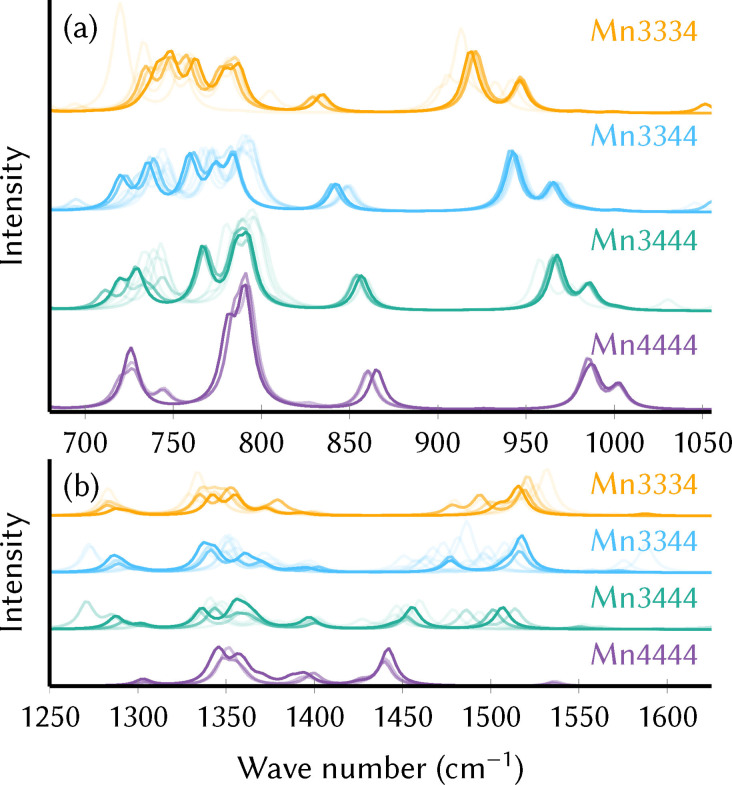
Superposition of all simulated IR spectra from different oxidation states (colors), local minima, and ligand configurations. The opacity of a line is determined by the Boltzmann weight of the corresponding minimum,^[17]^ with more probable minima drawn with more opaque lines. Mn3333 is not shown because^[17]^ it is prone to ligand dissociation and hence its energetics are not fully understood.

In order to monitor the oxidation‐state‐dependent shift of the V=O bands experimentally, we carried out in‐situ infrared attenuated total reflection (IR‐ATR) measurements during oxidation of the catalyst in water‐free acetonitrile solution in a custom‐made SEC cell (see Supporting Information Section S1.2 and Figure S3). For details on the preparation and purity of the compound, please see Supporting Information Section S1.3. Figure 3 shows the obtained IR‐ATR spectra in the spectral region between 920 and 1040 cm^−1^, i. e., where the characteristic V=O stretch bands are found. For the pristine precatalyst (Mn3344) that is present in the beginning of the measurement (0 min, blue line), the V=O stretch bands occur at 955 cm^−1^ and 970 cm^−1^, with a double peak very similar to the one seen in the simulated spectra. The shift of about 10 cm^−1^ compared to the calculated positions (Table 2) is probably due to the chosen level of theory. Interestingly, the IR spectrum recorded for solid MnV WOC (see Supporting Information Section S1.4 and Figure S4) is slightly shifted relative to the solution spectrum and matches the simulated spectra very well.

**Figure 4:**
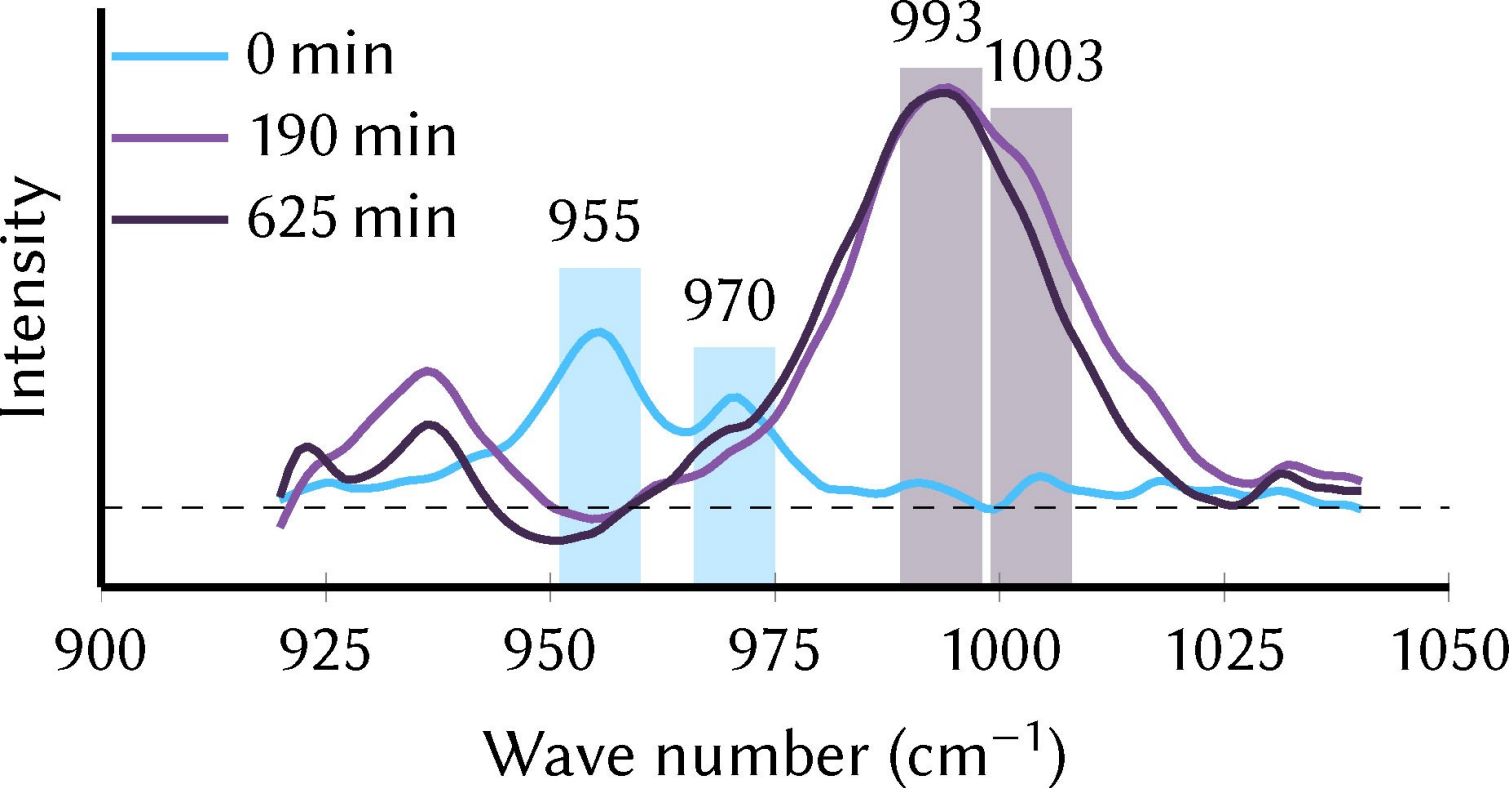
IR‐ATR spectra of the pristine catalyst Mn3344 in 0.1 M TBAPF_6_/acetonitrile prior to (0 min) and after initiating the oxidation (190 min and 625 min) at a potential of 1.25 V versus Fc/Fc^+^. The colored bars indicate the position of the V=O stretch double peak that was predicted to shift with the oxidation state.

After an oxidation period of 190 min, no more changes of the IR spectrum were evident and a steady state (i. e., complete oxidation close to the working electrode) was assumed. Applying a potential of 1.25 V (versus Fc/Fc^+^) leads to the formation of the oxidized species, Mn4444, clearly visible in the recorded square wave voltammogram (SWV, see Supporting Information Section S1.5 and Figure S5). The stronger V=O stretch band shifts by approximately 40 cm^−1^ from 955 to 993 cm^−1^. This shift is in good accordance with the theoretically predicted shift given in Table 2. The position of the weaker peak cannot be precisely extracted from the IR‐ATR spectrum, as the band becomes broader and simultaneously more intense. The observed change in band shape is hypothesized to be caused by accumulation or deposition effects of the oxidized Mn4444 species at the ATR waveguide surface, given the small cell volume within the thin‐film electrochemical cell. Based on the observation of a shoulder at 1003 cm^−1^, it appears that the corresponding vibration is shifted by only about 30 cm^−1^, which indicates that this vibration might be less affected by the change in oxidation state. However, the trends seen in Figure 4 certainly show that the oxidation state can be experimentally followed in‐situ based on the V=O vibration frequencies.

### Computed UV/Vis spectra

**Figure 5:**
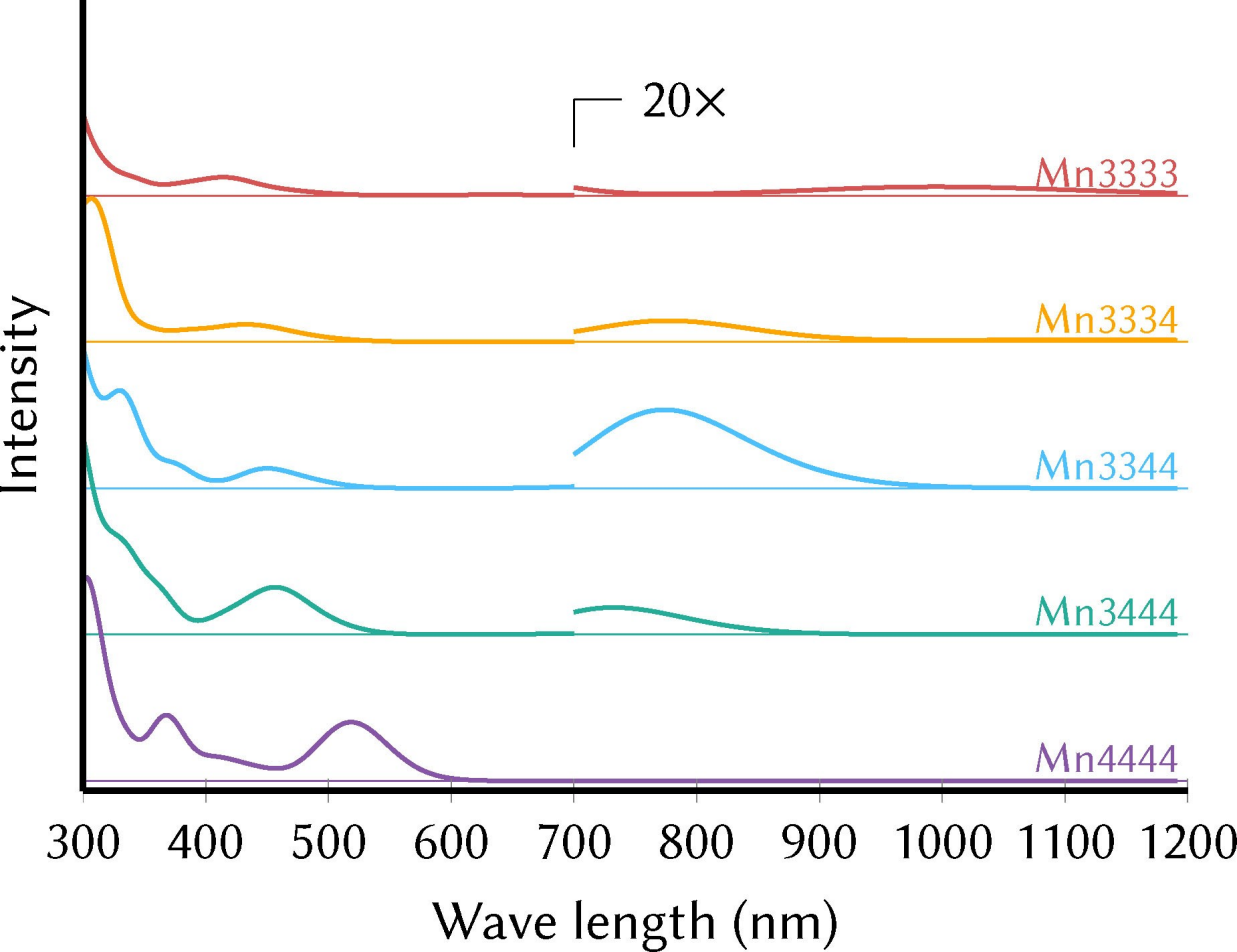
Simulated UV/Vis spectra of the different oxidation numbers of the precatalyst in the spectral region 300 to 700 nm.

Besides the infrared spectra, we also computed UV/Vis absorption spectra of all thermodynamically relevant local minima, using time‐dependent DFT (TD‐DFT) in solution. The simulated UV/Vis absorption spectra for the precatalyst in the different oxidation states are shown in Figure 5. In general, all oxidation states show strong absorption below 400 nm, although it appears that high oxidation states have more intense absorption in this region. At higher wavelengths, a band can be observed that has its maximum around 520 nm for Mn4444 and that is blueshifted significantly for the lower oxidation states (455 nm for Mn3444, 450 nm for Mn3344, 430 nm for Mn3334, 410 nm for Mn3333). Concomitant with the blueshift, this band also loses intensity at low oxidation states. Additional spectral information is provided by the bands at long wavelengths. Here, it is interesting to note that in the Mn4444 spectrum no transitions are observed above 600 nm, whereas in the other oxidation states low‐intensity transitions around 700–800 nm can be seen.

Like in the simulated IR spectra, we investigated how much the UV/Vis spectra vary with oxidation state, Jahn‐Teller configuration, and ligand configuration, as shown in Supporting Information Section S2.1 and Figure S6. It was found that these variations are somewhat larger than for the IR spectra, but the UV/Vis spectra nonetheless contain useful information about the oxidation state. The complete absence of absorption above 600 nm indeed indicates the Mn4444 oxidation state, and the 400–500 nm absorption band can be further used to roughly estimate which oxidation state is present in the reaction mixture.

**Figure 6:**
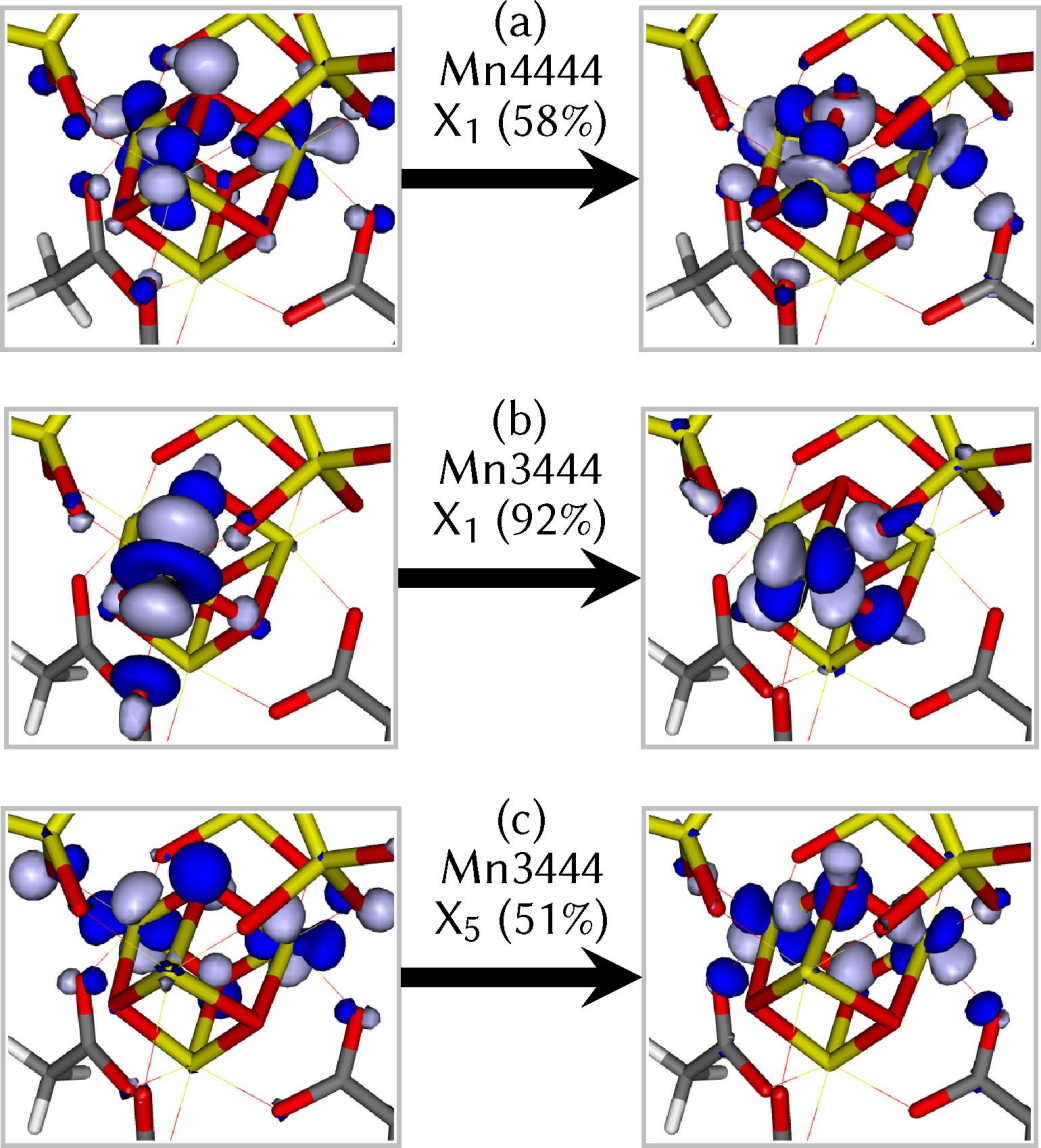
Natural transition orbitals for selected transitions in Mn4444 and Mn3444. The orbitals on the left indicate the hole from where excitation takes place, the right orbitals show where excitation is directed to. The labels give the oxidation state, excited‐state numbering (e. g., *X*_1_ is the first excited state), and contribution of the shown NTO pair to the transition.

More insight into the nature of the electronic excitations can be extracted by analyzing the associated state characters. For three selected states of Mn4444 and Mn3444, in Figure 6 we show the most important natural transition orbitals. It can be seen that the first excited state in Mn4444 (panel a) is a linear combination of local d→d transitions on the three Mn atoms adjacent to the vanadate ligand. This transition is responsible for the 520 nm peak shown in Figure 5. In contrast to this delocalized excitation, the lowest excitation in Mn3444 (panel b) is localized on the only Mn^III^ atom. This state is also a d→d transition, but the hole orbital is the $d_{z^{2}}$ that became occupied during the Mn^IV^/Mn^III^ reduction. The orbital of the excited electron is the corresponding $d_{x^{2}-y^{2}}$ orbital. The gap between these two arises only from the Jahn‐Teller distortion around the Mn^III^ and consequently is rather small. This explains the presence of the absorption band at about 750 nm for Mn3444 in Figure 5. Panel (c) shows the fifth excitation in Mn3444, which is the one that most closely resembles the first excitation of Mn4444, with the difference that only two Mn^IV^ atoms contribute.

**Figure 7:**
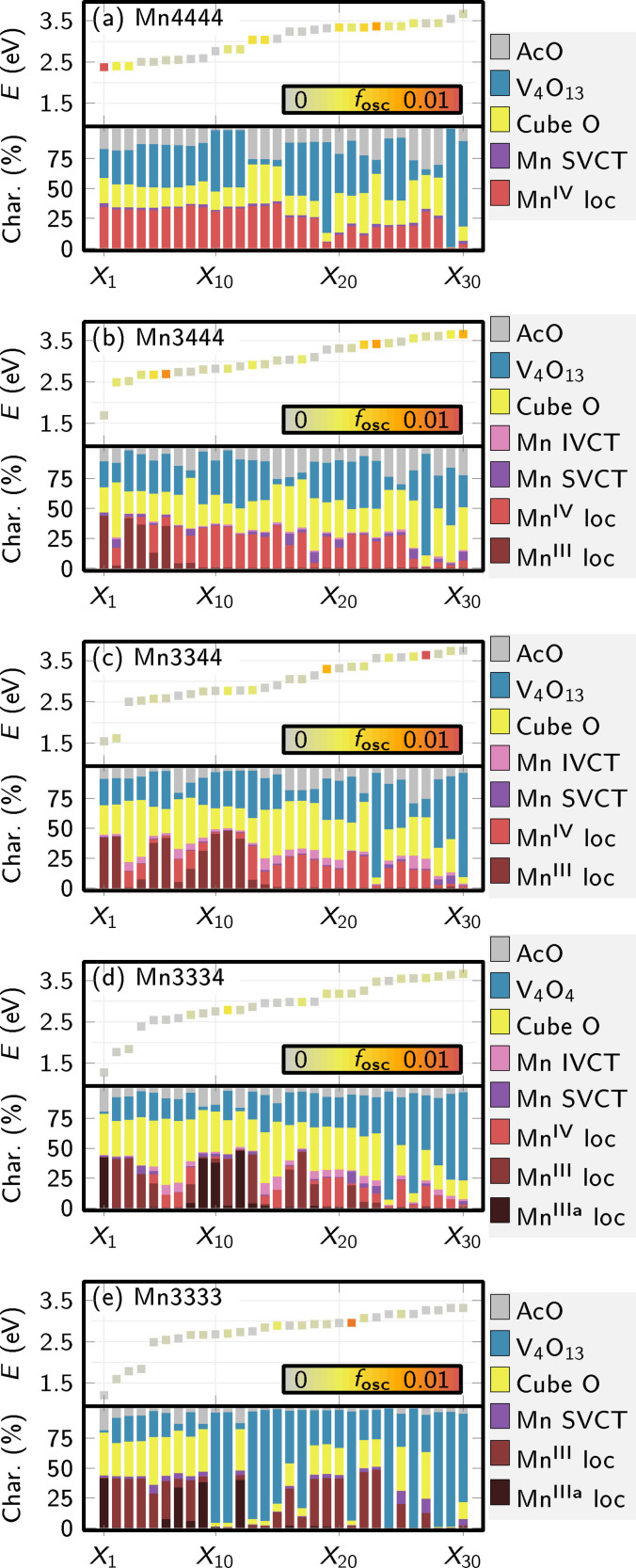
Excited‐state and charge transfer analysis plot for the first 30 excited states of all oxidation states. For each oxidation state, the upper panel shows excitation energy and oscillator strengths. The lower panel shows the charge transfer characters of all states, based on the excitation classes on the right. loc: local d→d excitations on Mn atoms (Mn^IIIa^ indicates the apical Mn atom); SVCT: same‐valence charge transfer (between Mn atoms of the same oxidation state); IVCT: intra‐valence charge transfer (between Mn atoms of different oxidation states); Cube O: excitation contributions involving the cube oxygen atoms; V_4_O_13_: excitation contributions involving the vanadate; AcO: excitation contributions involving the acetates (more details in Supporting Information Section S2.2).

A more quantitative and orbital‐free analysis of the excitations in the MnV WOC was carried out by means of a fragment‐based charge transfer analysis,^[17]^ explained in Supporting Information Section S2.2 and Figures S7 and S8. The results for the 30 lowest excitations of all five oxidation states are condensed in Figure 7; the analysis of the 100 excitations used to generate the spectra in Figure 5 is in Supporting Information Section S2.3 (Figures S9 to S14). The first data point (*X*_1_ state) of Figure 7a represents the transition that is shown in Figure 6a for the Mn4444 oxidation state. The charge transfer analysis shows that formally, this transition is partially delocalized over the entire molecule (including oxygens, vanadate, and acetates), but the most important part is the large “Mn^IV^ loc” contribution that indicates a local Mn^IV^ d→d transition. Most of the states shown in Figure 7 are dominated by such local transitions. In contrast, all computed higher excitations (above 3.5 eV) are due to transitions on the vanadate ligand (Supporting Information Section S2.3), which provide limited information on the oxidation state of the Mn atoms. Consequently, the low‐energy part of the spectrum (below 3.5 eV, i. e., above 350 nm) – dominated by Mn‐centered excitations – is most relevant for the identification of the oxidation state.

Figure 7 also helps understanding how the low‐energy region of the UV/Vis spectrum can deliver information on the Mn oxidation states. The lowest transitions localized on Mn^IV^ atoms (“Mn^IV^ loc”) are positioned at about 2.4–2.5 eV (500–520 nm). In contrast, each Mn^III^ atom contributes one low‐energy transition at about 1.1–1.7 eV (730–1130 nm) due to a transition similar to the one shown in Figure 5b. Thus, only Mn^III^ atoms can produce any absorption above about 600 nm. However, as the Mn^III^ orbitals are rather localized, the d→d transitions in Mn^III^ tend to follow the LaPorte rule and only exhibit very small intensities. Such low‐intensity and low‐energy absorption was already found by Schwarz et al.,^[8]^ (reproduced in Supporting Information Figure S15), who observed a very weak (150 M^−1^cm^−1^) absorption band at 680–850 nm for the pristine precatalyst (Mn3344). Notably, these authors originally assigned this transition to an intra‐valence charge transfer (IVCT), as often observed in mixed‐valence poly‐nuclear metal complexes. However, our analysis does not identify any IVCT and SVCT (same‐valence charge transfer) transitions within the computed states, up to about 4.5 eV. Instead, this low‐energy band should be assigned to local d→d transitions in Mn^III^ atoms.

### Experimental UV/Vis spectra

**Figure 8:**
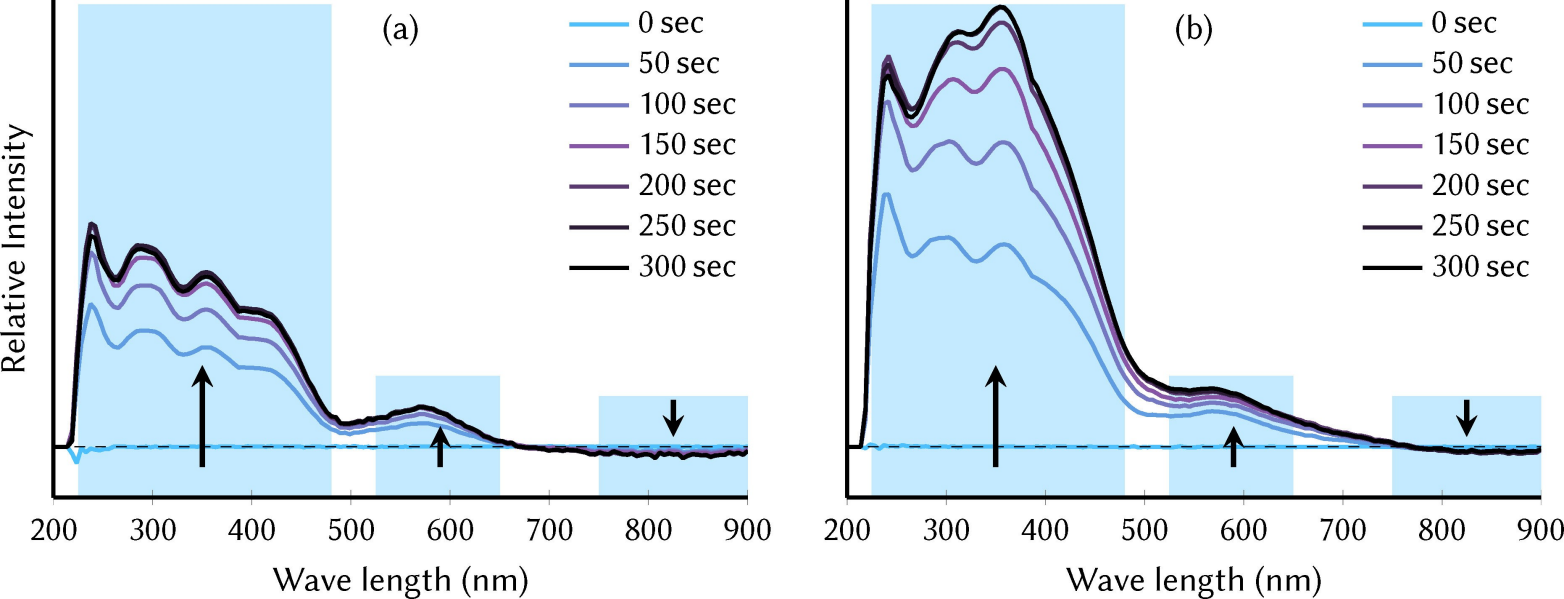
UV/Vis differential spectra of the pristine catalyst Mn3344 (250 μ M, used a background) in acetonitrile containing 0.1 M TBAPF6 prior to (0 sec) and during oxidation (>0 sec) at a potential of (a) 0.4 V and (b) 1.3 V versus Fc/Fc^+^. The colored bars indicate the three general spectral regions discussed in the text.

To observe the UV/Vis absorption of the different oxidation states of the MnV WOC, we measured in‐situ differential spectra in a SEC cell, using potentials of 0.4 V and 1.3 V versus Fc/Fc^+^. The resulting differential spectra are shown in Figure 8a and b, respectively. As indicated by the SWV (see Supporting Information Section S1.5), the former potential is expected to produce the Mn3444 oxidation state, whereas the latter potential further oxidizes the MnV WOC to the Mn4444 oxidation state. In the figure, the initial zero lines correspond to the pristine catalyst in the Mn3344 oxidation state. In the course of the measurements, three bands evolve in the difference spectrum while the complex is oxidized to the Mn3444 or Mn4444 oxidation state. After 300 seconds, no further spectral changes are observed, suggesting that a steady state of oxidation of the substrate at the mesh electrode was reached. For both applied potentials, the final spectrum shows that an intense band in the 220–480 nm region appears upon oxidation (i. e., absorption in this region becomes more intense compared to the background signal). The band shapes are not in complete agreement with the simulated UV/Vis spectra (probably due to the limitations of the TD‐DFT method in describing high‐lying excited states) but allow us to nevertheless assign this high‐energy band primarily to vanadate transitions. The increase of absorption in this band during oxidation is in line with the simulations, which show a more intense UV band for Mn3444 or Mn4444 than for Mn3344 (see Figure 5). This band also features the most distinct differences between the two applied potentials: at 0.4 V, the most intense absorption is observed around 250 nm, whereas at 1.3 V the absorption at 350 nm is stronger. This indicates slight differences in the vanadate transitions in both oxidation states, not resolvable with the TD‐DFT calculations. It is interesting to note that the spectra at 0.4 V closely resemble the spectrum recorded after 50 sec at 1.3 V, indicating that a measurable amount of Mn3444 is formed before it is further oxidized to Mn4444.

A second, less intense band is arising in Figure 8 in the 520–650 nm region. This band can be assigned to the lowest bright excited states of the oxidized species, which are linear combinations of d→d transitions of the non‐apical Mn^IV^ atoms (as in Figure 6a). As shown in Figure 5, this peak is most intense in the Mn4444 oxidation state, matching the experimental finding that absorption in the 520–650 nm region increases during oxidation and is more intense if a larger potential is applied.

Very interestingly, the SEC experiment also finds that intensity in the 750–900 nm region decreases during oxidation. This is in very good agreement to the computed spectra (Figure 8), which show very clearly that Mn3344 absorbs more strongly at very long wavelengths above 680 nm than Mn3444, and Mn4444 does not have any transitions in this region. Hence, the decrease in absorption in the 750–900 nm region is directly correlated to the decrease in the number of Mn^III^ atoms in the beam path near the working electrode. Again, the computationally predicted changes in the spectra are nicely recovered by the in‐situ experiments, showing that extracting information about the oxidation state of the MnV WOC is feasible with UV/Vis spectroscopy.

## Conclusions

In this study, a complementary computational and experimental approach to obtain IR and UV/Vis signatures of the manganese‐based water oxidation catalyst [(Mn_4_O_4_)(V_4_O_13_)(OAc)_3_] ^3− [8]^ was adopted. As this catalyst passes through multiple oxidation states during catalysis, the spectroscopic characterization of the involved oxidation states is an important step in the elucidation of the activation and catalytic mechanism. Our results found that the IR spectrum contains a few marker bands that can be used to reliably track those. One set of bands is in the 1450–1550 cm^−1^ region, where OCO asymmetric stretch modes are located at about 1450 cm^−1^ if bonded to Mn^IV^ and at about 1550 cm^−1^ if bonded to Mn^III^. A second set of bands is located at 830–1000 cm^−1^ and consists of V=O and VOV stretch modes that shift predictably by 20 cm^−1^ for each oxidation step. No other vibrations of the molecule are present in these two regions, allowing for direct identification of the oxidation state. We also investigated UV/Vis spectra, which might be easier to measure in‐situ while the catalysis is taking place. We found weak d→d transitions in the 700–900 nm range that can be used to probe the presence of Mn^III^ atoms. Furthermore, the absorption band/shoulder around 500—600 nm undergoes a shift towards higher energies and lower intensities upon reduction.

The marker bands identified in IR and UV/Vis spectra were shown to be relatively robust with respect to the molecular structure, based on multiple spectra simulations for different oxidation states, orientations of the Jahn‐Teller axes of Mn^III^, and the type of attached ligands. Whereas the two latter modifications did not have a strong influence on the spectra, the oxidation state does lead to clear and systematic changes in the spectrum. In‐situ spectroelectrochemical measurements of the IR and UV/Vis spectra nicely agree with the predicted spectral changes upon oxidation from the [Mn^III^Mn${}_{3}^{\mathrm{IV}}$ ] to the [Mn${}_{4}^{\mathrm{IV}}$ ] oxidation state. Future experimental work will look at further oxidation states and at spectral changes induced by ligand exchange reactions. This will allow to advance understanding on the catalytic activity of this water oxidation complex.

## Experimental Section

### Computational Details

All stable local minima of all oxidation states (described in Ref. [17]) were optimized and a vibrational analysis carried out with Gaussian 16,^[19]^ the BP86 functional,[^20^, ^21^] def2‐SVP^[22]^ for C and H, and def2‐TZVP^[22]^ for Mn, V, and O. The IEFPCM implicit solvent model^[23]^ (acetonitrile) and GD3 dispersion correction^[24]^ were used. No scaling was applied to the computed vibrational frequencies. The simulated IR spectra were obtained by convolution of the results with a Lorentzian with full‐width at half‐maximum of 10 cm^−1^.

UV/Vis absorption spectra were computed using ORCA 4.2.1[^25^, ^26^] at all stable local minima. We used the Tamm‐Damcoff approximation, the CAM‐B3LYP[^27^, ^28^] functional, ZORA‐SVP[^22^, ^29^] for C and H, and ZORA‐TZVP[^22^, ^29^] for Mn, V, and O. Implicit solvation was included with C‐PCM (acetonitrile),^[30]^ and the RIJCOSX approximation^[31]^ was used to speed up the calculations. Spectra were computed from 100 excited states by convolution with a Gaussian with full‐width at half‐maximum of 0.3 eV. Fragment‐based charge transfer analysis was performed with the TheoDORE package,^[32]^ for which we implemented the analysis of spin‐unrestricted calculations.

### In‐situ IR Methods

In‐situ IR‐ATR measurements during oxidation of the catalyst were performed using a Fourier transform infrared spectrometer (Alpha I, Bruker Optics GmbH, Ettlingen, Germany) operated with a room temperature DLaTGS (deuterated L‐alanine doped triglycine sulfate) detector and an ATR accessory comprising a single‐bounce diamond ATR crystal (Platinum ATR, Bruker Optics GmbH, Ettlingen, Germany). Data were recorded in the range of 400–4000 cm^−1^ at a spectral resolution of 2 cm^−1^ averaging 64 scans for each spectrum resulting in a measurement time of 125 s per spectrum. Spectra were recorded over a period of 625 min. A volume of 3 mL of sample solution (6 mM MnV WOC in 0.1 M TBAPF_6_ solution in acetonitrile) was applied using a custom‐made liquid cell (Teflon) sealed against the ATR module (Supporting Information Section S1.2). A background spectrum of the solution was recorded also averaging 64 scans prior to inserting the sample solution.

In the in‐situ IR‐ATR measurements, the oxidation of MnV WOC from oxidation state Mn3344 to Mn4444 was performed using a CHI842B potentiostat (CH Instruments, Austin, TX, USA) and a three‐electrode setup with a 3 mm (diam.) glassy carbon (GC) electrode as working electrode, an Ag wire as reference electrode, and a Pt wire as counter electrode. All potential values are reported versus Fc/Fc^+^. Prior to the spectroelectrochemical experiments, the GC electrode was polished using red Technotron cloth (LECO, St. Joseph, MO, USA) and an aluminum oxide suspension (LECO, St. Joseph, MO, USA); then, the electrode was cycled 10 times in 0.5 M aqueous H_2_SO_4_ between −1 V and 1 V versus Hg/Hg_2_SO_4_ to remove impurities. For the oxidation of 6 mM MnV WOC dissolved in 0.1 M TBAPF_6_/acetonitrile, a potential of 1.25 V versus Fc/Fc^+^ was applied. Prior to applying the potential, the GC electrode was positioned at the ATR crystal establishing an electrochemical thin‐film cell. The developed measurement cell was constructed in a way that a spacer ensured that the working electrode was located 3 mm above the ATR crystal. The volume between the GC electrode and the ATR crystal was approximately 20 *μ*L. The electrochemical oxidation of the compound and the IR‐ATR measurements were started simultaneously.

A SWV was recorded in bulk solution using a Gamry 1010B potentiostat (SWV parameters: frequency of 25 Hz, pulse amplitude of 25 mV, potential step of 4 mV in a range between −0.65 V and 1.25 V versus Fc/Fc^+^).

For pre‐treatment of the electrodes, 0.5 M H_2_SO_4_ solution (≥98 %, per analysis, VWR Chemicals, VWR International GmbH, Darmstadt, Germany) was prepared with high purity water (18.1 MΩcm, Barnstead Nanopure – Thermo Fisher Scientific, Dubuque, USA). As electrolyte salt, tetra‐*n*‐butylammoniumhexafluorophosphate (TBAPF_6_, 98 %, Alfa Aesar, ThermoFisher GmbH, Kandel, Germany) was dissolved in water‐free acetonitrile (anhydrous, 99.8 %, Sigma‐Aldrich, St. Louis, USA). All commercially available chemicals were used as purchased without further purification.

### In‐situ UV/Vis Methods

For the in‐situ SEC UV/Vis experiment, we used a batch cell type cuvette with 1 mm path length, a platinum mesh as working electrode, a platinum wire as counter electrode, and an Ag/Ag^+^ (RE‐7) non‐aqueous reference electrode composed of a silver wire immersed in acetonitrile containing 0.1 M tetrabutylammonium perchlorate and 0.01 M AgNO_3_, separated from the outer solution by a Vycor glass frit. As electrolyte solution, we used a water‐free acetonitrile solution containing 0.1 M of TBAPF_6_ as supporting electrolyte and 250 μM of the MnV WOC. The potential was applied at 0.4 V or 1.3 V versus Fc/Fc^+^ using a Gamry 1010B potentiostat. The slight difference in potential between the IR‐SEC (1.25 V) and UV/Vis‐SEC (1.3 V) measurements is due to the different experimental setups and concentrations, as well as expected small shifts when employing pseudo‐reference electrodes.^[33]^ The UV/Vis spectra have been measured using an Avantes μs‐UV/Vis‐Spectrometer (AvaSpec‐ULS2048CL) between 220 and 900 nm with an integration time of 0.9 ms; 300 spectra were collected and averaged. The cell solution (containing electrolyte and MnV WOC) was subtracted as background reference.

## Author contribution statement

All computations were conceived by SM and LG, carried out by MH, AA, and SM, and analyzed by SM. IR‐ATR‐SEC experiments were designed, performed and analyzed by SK, JK, and RS. UV/Vis‐SEC experiments were designed, performed, and analyzed by IT. Static IR spectra were measured by IT. SWV measurements were done by IT and JK. The manuscript draft was written by SM, with contributions by LG, SK, JK, and IT. RS, CK, BM, CS proofread the article. All authors have given approval to the final version of the manuscript.

## Supporting Information

Supplementary results on infrared spectroscopy (peak assignment of experimental spectrum, experimental setup for in‐situ IR‐ATR SEC measurements, influence of solvation, square wave voltammetry) and UV/Vis spectra (influence of local minima, fragmentation scheme for charge transfer analysis, electronic state characterization, experimental static UV/Vis spectrum).

## Conflict of interest

The authors declare no conflict of interest.

## Supporting information

As a service to our authors and readers, this journal provides supporting information supplied by the authors. Such materials are peer reviewed and may be re‐organized for online delivery, but are not copy‐edited or typeset. Technical support issues arising from supporting information (other than missing files) should be addressed to the authors.

Supporting Information
