# Supplementary material for: Spectral Signatures of Oxidation States in a Manganese‐Oxo Cubane Water Oxidation Catalyst
Source: Chemistry. 2021 Oct 15;27(68):17078–86. doi: 10.1002/chem.202102583 (PMC9293465; doi:10.1002/chem.202102583)
Supplement: Supplementary file 1 — Supporting Information [file CHEM-27-17078-s001.pdf]

Chemistry–A European Journal

Supporting Information

Spectral Signatures of Oxidation States in a Manganese-Oxo Cubane Water Oxidation Catalyst

Sebastian Mai,* Sarah Klingler, Ivan Trentin, Julian Kund, Marcus Holzer, Anastasia Andreeva, Robert Stach, Christine Kranz, Carsten Streb, Boris Mizaikoff, and Leticia González*

Contents

S1 Infrared spectra **2**
S1.1 Peak assignment 2
S1.2 Experimental Setup for in-situ IR-ATR Spectroelectrochemical Measurements 4
S1.3 Synthesis and purity characterization of the pristine catalyst 5
S1.4 Influence of the solvent on the spectral position of the V=O vibration 5
S1.5 Square wave voltammetry 6

S2 UV/Vis spectra **7**
S2.1 Influence of local minimum on UV/Vis spectra 7
S2.2 Fragmentation for electronic state characterization 8
S2.3 Electronic state characterization 9
S2.4 Experimental UV/Vis spectrum 13

S1 Infrared spectra

S1.1 Peak assignment

Experimental IR spectra of solid [(nBu$_4$N)$_3$(Mn$_4$O$_4$)(V$_4$O$_{13}$)(OAc)$_3$] (i.e., the pristine precatalyst in the Mn3344 oxidation state) were recorded using a Fourier-transform infrared (FT-IR) spectrometer (Alpha II-Platinum, high-pressure version, Bruker Optik GmbH, Ettlingen, Germany) equipped with a monolithic diamond crystal. The spectrum was collected at a resolution of 4 cm^{-1}, averaging 24 spectra per measurement. A spectrum is shown in Figure S1 together with an assignment of the vibrations based on the computational IR results. The figure gives an overview over the different regions of the spectrum.

Starting at high wave numbers, the compound shows a triple band in the CH stretch region around 2800–3000 cm^{-1}, coming from the acetate methyl groups and the methyl and methylene groups of the tetra-n-butylammonium counterions.

In the 1300–1600 cm^{-1} range, a total of 15 vibrations of the MnV WOC are located, where each acetate ligand contributes 5 vibrations. These are the symmetric and asymmetric OCO stretch modes with strong absorption and three deformation modes per methyl group. It is likely that at least one of the peaks in this region is due to absorption of the NBu_4^+ counterions, as the experimental spectrum contains bands (at 1459 and 1480 cm^{-1}) that are not present in the simulated IR spectrum of the Mn3344 oxidation state (see below).

Between 900 and 1000 cm^{-1} we could locate a total of 13 vibrations of the MnV WOC, with 4 very intense V=O stretch modes and 9 weak modes from the three acetates, one CC stretch and two methyl rocking modes per acetate.

In the 700–900 cm^{-1} range only three VOV stretch modes and three MnOV stretch modes are located, all of which are very intense. No further vibrations (even weak transitions) are observed in this region.

Below 700 cm^{-1}, a congested region with many different vibrations is encountered. Strong transitions tend to be due to combinations of cubane breathing modes, MnOV and VOV stretch modes, or OCO deformation/rocking modes. About 30 of the 132 normal modes of MnV-Ac$_3$ can be found in the 400–700 cm^{-1} region.

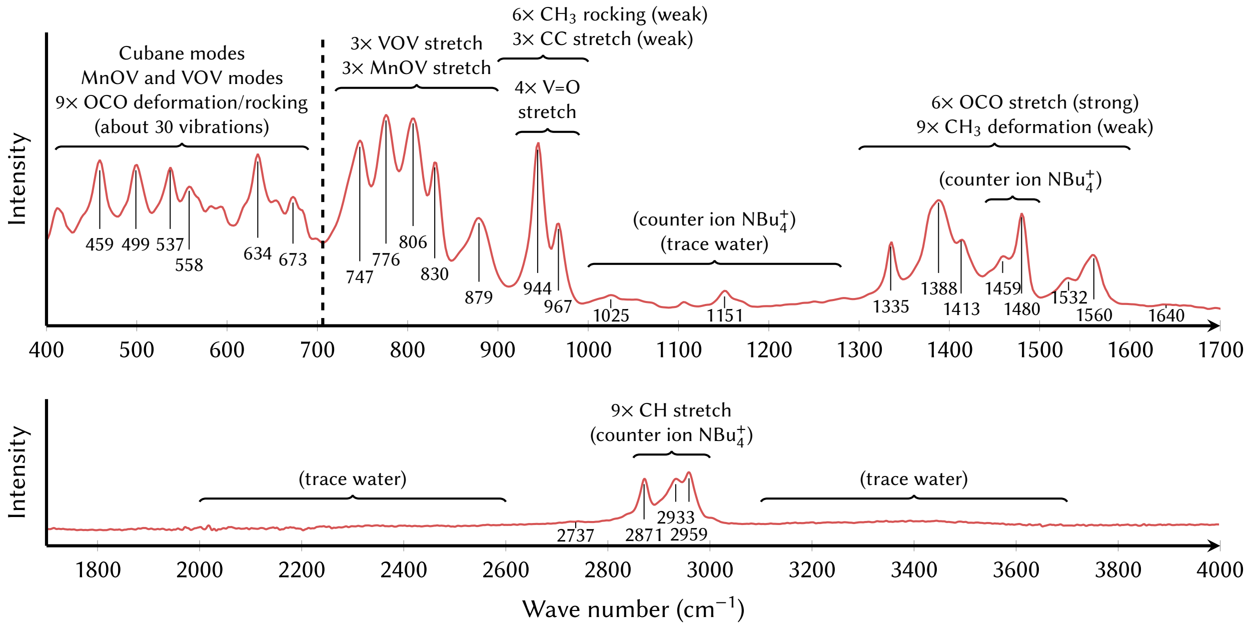

Figure S1: Experimental IR spectrum of the pristine catalyst (MnV-Ac$_3$ in the Mn3344 oxidation state) and assignment of vibrational modes based on the computed IR spectra. The dashed line separates the hard-to-analyze fingerprint region from the well-structured rest of the spectrum. The number of MnV WOC vibrations to the right of the dashed line are explicitly enumerated in the labels.

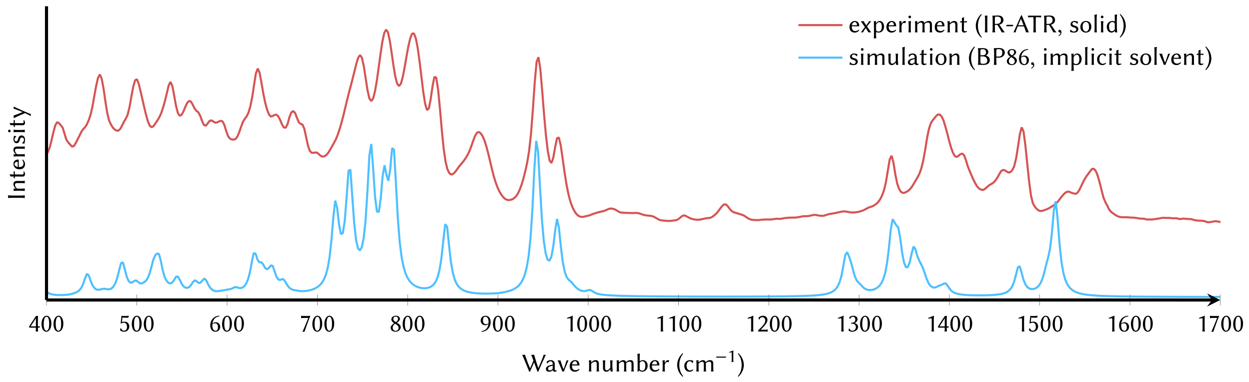

Figure S2: Comparison of the IR spectra shown in Figures S1 and Figure 2 in the main manuscript. The CH stretch region is omitted because the experimental spectrum is dominated by signals from the counter ion NBu_4^+ there.

In Figure S2, we compare the IR spectra of the Mn3344 oxidation state shown in Figures S1 (experimental, solid) and Figure 2 (computation, implicit solvation) in the main manuscript. We observe a very good qualitative agreement between the two. The three peaks around 500 cm^{-1} are slightly shifted in energy, but the complex band at 630 cm^{-1} is reproduced well. The MnOV and VOV bands around 700–800 cm^{-1} that give rise to a prominent quadruple peak in the experimental spectrum are split into five peaks and shifted to lower energy in the computed spectrum. Likewise, the isolated VOV peak (exp: 880 cm^{-1}, theo: 840 cm^{-1}) is shifted and narrower in the computation. This is because the simulated spectrum uses a fixed line shape (Lorentzian with 10 cm^{-1} full width at half maximum), whereas in the experiment different bands have different widths. The V=O stretch marker bands at about 950 cm^{-1} are very well reproduced by the simulations. The different peaks in the 1300–1600 cm^{-1} region are shifted to lower energies in the computations, although the band shape matches very well. Note that the peak at 1480 cm^{-1} in the experimental spectrum is not due to the MnV WOC, but to the NBu_4^+ counterion, and is therefore not included in the computed spectrum.

Most deviations in the band shapes of experimental and simulated spectrum can be explained by the absence of either the three tetra-*n*-butylammonium counterions or the other local minima of the Mn3344 oxidation state in the simulated spectrum shown in Figure S2. Furthermore, it cannot be excluded that a small amount of trace water is present in the experimental spectrum. The shift in some of the computed bands is most probably due to the electronic structure level of theory (exchange-correlation functional, basis set, implicit solvation). However, given the challenging electronic structure problem of the polyoxometalate catalyst, the simulated IR spectra are very good.

S1.2 Experimental Setup for in-situ IR-ATR Spectroelectrochemical Measurements

For in-situ spectroelectrochemical IR-ATR experiments, a custom-made liquid cell was used generating a thin-film electrochemical cell with the ATR crystal at the bottom of the cell. The setup shown in Figure S3a comprises a cylindrical bottom component (D) made from Teflon with an opening, facilitating ATR measurements. The cell is closed with a lid (E) providing three ports for inserting electrodes. The glassy carbon electrode (B in panel (a), GC in panel (b)) encloses a small sample volume of approximately 20 μL, realizing a thin-film electrochemical cell. The Pt wire (C) serves as counter electrode, while an Ag wire acts as reference electrode (A). In Figure S3b, a schematic of the measurement principle is shown, displaying the thin-film electrochemical cell combined with the IR-ATR assembly.

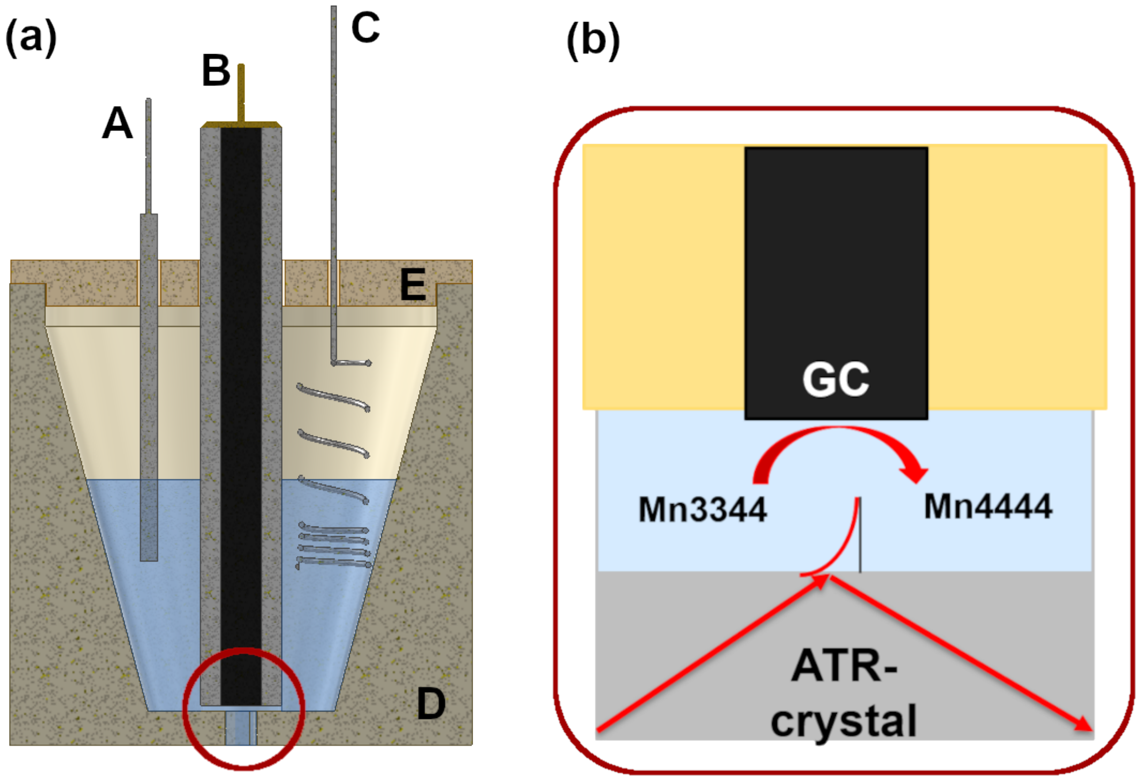

Figure S3: Schematic of (a) the custom-made electrochemical thin-film cell, and (b) the measurement principle of the spectroelectrochemical IR-ATR experiments. For explanations of the labels A, B, . . . , see the text.

S1.3 Synthesis and purity characterization of the pristine catalyst

We repeat here from Ref. 1 the synthetic procedure to prepare the pristine catalyst, $(n\text{-}Bu_4N)_3[(Mn_4O_4)(V_4O_{13})(OAc)_3]\times 3H_2O$. One equivalent of $(n\text{-}Bu_4N)_4[V_4O_{12}]\times 4H_2O$ (401.4 mg, $2.79 \cdot 10^{-4}$ mol), four equivalents of $Mn(OAc)_3\times 2H_2O$ (299.3 mg, $1.12 \cdot 10^{-3}$ mol), and one equivalent of $(n\text{-}Bu_4N)MnO_4$ (101.6 mg, $2.81 \cdot 10^{-3}$ mol) were dissolved in 16 ml acetonitrile. An aqueous $(n\text{-}Bu_4N)OH$ solution (200 μl, 20 wt.-%) was added and the solution was vigorously stirred for one hour at room temperature. Diffusion of diethyl ether into the reaction mixture gave dark brown cubic single crystals, suitable for X-ray diffraction studies. Yield: 237.9 mg ($1.49 \cdot 10^{-4}$ mol, 53.3% based on V).

Elemental analysis for $Mn_4V_4O_{26}N_3C_{54}H_{123}$ in wt,.% (calcd.):[1] C 39.14 (39.21), H 7.38 (7.50), N 2.48 (2.54), Mn 13.65 (13.29), V 13.12 (12.32).

The X-ray structure, reported in Ref. 1, can be found in the data base of the Cambridge Crystallographic Data Centre (CCDC), ID 898055.

Characteristic IR bands (in cm^{-1}, see also Figure S1): 3437 (vb), 2959 (vs), 2933 (vs), 2871 (s), 2359 (w), 2339 (w), 1560 (s), 1480 (vs), 1388 (vs), 1335 (s), 1151 (m), 1025 (w), 967 (s), 944 (vs), 879 (m), 830 (vs), 806 (vs), 776 (vs), 747 (s), 634 (m), 537 (m), 499 (m), 459 (m). The IR spectrum matches very well with the IR bands previously reported together with the X-ray structure and elemental analysis.[1]

S1.4 Influence of the solvent on the spectral position of the V=O vibration

Figure S4 compares the IR bands of the V=O vibrations in the solid state spectrum with the ones in the spectrum recorded in acetonitrile solution. As can be seen, in solution the most intense V=O stretching band is shifted by 12 cm^{-1} (from 944 cm^{-1} to 956 cm^{-1}). The smaller peak shifts by 4 cm^{-1} (from 967 cm^{-1} to 971 cm^{-1}).

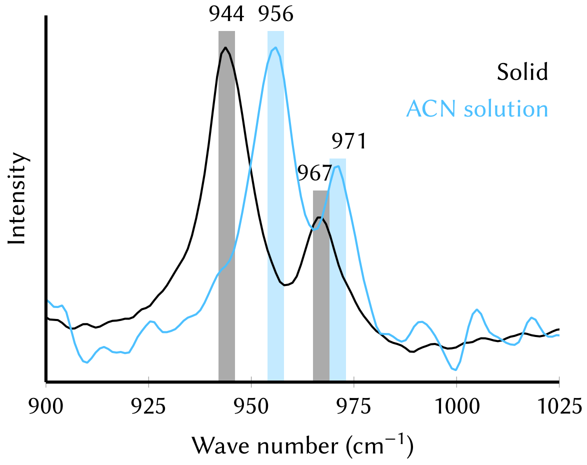

Figure S4: Comparison of the IR-ATR spectra of solid Mn3344 (black) with solvated Mn3344 (6 mM) in 0.1 M $TBAPF_6$/ACN solution (blue). The IR spectra were normalized to the maximum of the V=O stretching vibration.

S1.5 Square wave voltammetry

Figure S3 shows a typical SWV of the MnV WOC in bulk solution. The SWV reveals two characteristic peaks of the initial complex Mn_4V_4 at -0.03 V (Mn3344/Mn3444) and $+0.97$ V (Mn3444/Mn4444), which were previously shown by Schwarz et. al.[1] Note that previous cyclic voltammetry measurements[1] have shown that these two redox steps are quasi-reversible.

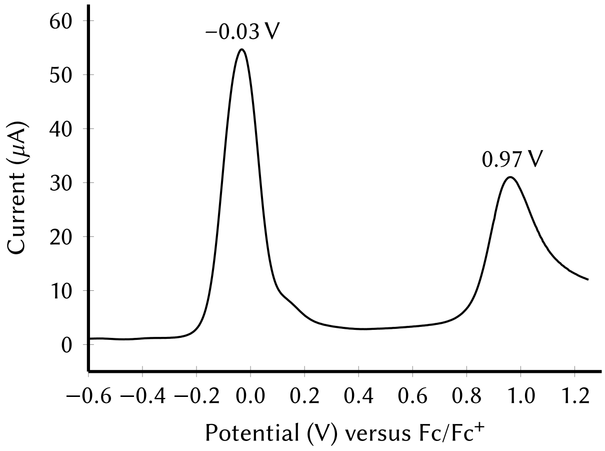

Figure S5: SWV of 6 mM MnV WOC in 0.1 M $TBAPF_6$/ACN performed in bulk solution. SWV parameters: $f = 25$ Hz, $E_{SW} = 25$ mV, $E_{step} = 4$ mV; working electrode: glassy carbon; counter electrode: Pt wire; reference electrode: Ag wire.

S2 UV/Vis spectra

S2.1 Influence of local minimum on UV/Vis spectra

Figure S6 shows a superposition of all simulated UV spectra from all oxidation states, all local minima due to Jahn–Teller effects, and all considered ligand configurations (three acetates or two acetates and H_2O/OH^-). The figure very nicely shows how the UV spectra are affected by these properties. The variations in the spectra due to Jahn–Teller effects and ligand configurations are larger than in the IR spectra discussed above, but nonetheless some interesting trends can be observed. Most importantly, the figure evidences that Mn4444 does not absorb at all above 600 nm, independent of ligand status. On the contrary, lower oxidation states always exhibit very weak transitions in this range. Unfortunately, there is significant variation in the low-energy absorption with Jahn–Teller configuration and ligand status, so this absorption cannot unambiguously identify the actual oxidation state. Some further information could be obtained by the absorption band in the 400–600 nm range, which shows a rather consistent shift with oxidation state.

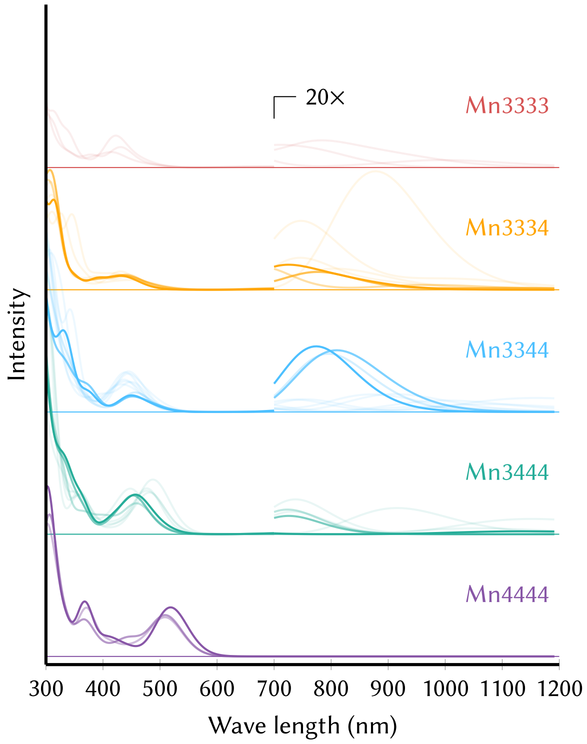

Figure S6: Superposition of all simulated UV spectra. The different sets of spectra correspond to the different oxidation states. For each oxidation state, multiple lines are drawn for the different local minima (due to the Jahn–Teller effect) and for the different ligand configurations. The opacity of a line is determined by the Boltzmann population of the corresponding structure, where conformations with low free energies (high Boltzmann weight) are drawn with solid lines and high-energy conformations with transparent lines.

S2.2 Fragmentation for electronic state characterization

The electronically excited states in the UV/Vis spectra were analyzed by means of a fragment-based charge transfer analysis performed with the TheoDORE program. For the analysis, the molecule was divided into 15 small fragments (8 fragments for the cubane, 3 acetate ligands, 3 VO_4 units, 1 VO unit), as shown in Figure S7 (left). The charge transfer analysis then yields for each state a 15×15 matrix that describes from which fragment and to which fragment electron density is excited. Because inspection showed that most matrix elements of these 15×15 matrices are vanishingly small, we simplified these 15^2 values to the 12 excitation classes shown in Figure S7 (right). Figure S8 shows a similar matrix with the excitation classes used in Figure 6 in the main text.

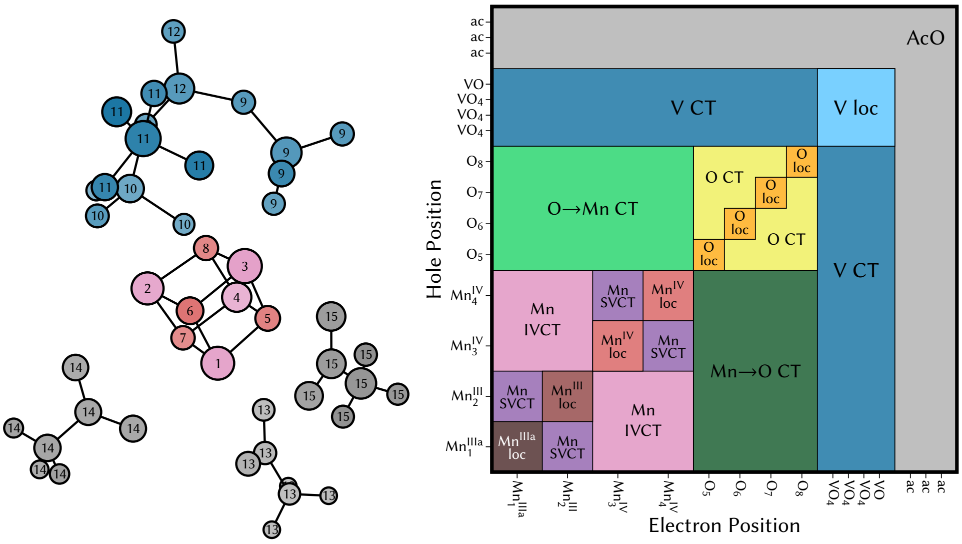

Figure S7: Scheme explaining the fragmentation of MnV-Ac₃ for the charge transfer analysis (left). The molecule was divided into 15 fragments (each Mn, each cubane O, each VO_4 group, the central VO group, each acetate). In this way, the charge transfer analysis produces a 15×15 matrix for each excited state. Based on analysis of this data, the excitation classes (right) were defined. Here we show an example for the Mn3344 oxidation state with one Jahn–Teller axis at the apical Mn atom. Depending on oxidation state and Jahn–Teller axes, the lower left block of the matrix is adapted. As an example how to read this scheme, the excitation class "O→Mn CT" includes all excitations from any atomic orbital on any cubane oxygen atom to any orbital on any manganese atom.

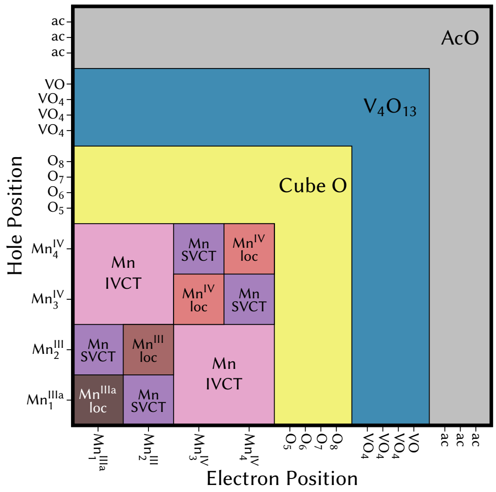

Figure S8: Like Figure S7, but with the simpler excitation classes scheme used for Figure 6 in the main manuscript.

S2.3 Electronic state characterization

Figures S9 to S13 present excitation energies, oscillator strengths, and charge transfer composition for all 100 states of all five oxidation states of MnV-Ac_3 (only most stable local minima). In Figure S9, the excited states of the Mn4444 oxidation state are analyzed. The top panel shows the energy and oscillator strength of the 100 computed states. As can be seen, the first excited state at 2.4 eV shows a significant oscillator strength. This state is responsible for the 520 nm absorption band in the UV/Vis spectrum of that oxidation state. Other strongly absorbing states can only be found around 3.5 eV and more so above 4.0 eV. The charge transfer analysis shows that all low-energy excitations are due to local Mn^{IV} excitations ($d \to d$ transitions), or linear combinations of such local excitations. No charge transfer transitions between different Mn atoms (same-valence charge transfer, SVCT) were found. Based on this, one can estimate the idealized number of Mn-centered transitions for this oxidation state to be 24, due to having four Mn atoms that each have three occupied and two virtual d orbitals (so each Mn atom contributes six $d \to d$ transitions). The calculations also show that only some Mn $d \to d$ transitions are bright, whereas most are dark. This can be explained by the LaPorte rule, which states that $d \to d$ transitions in an octahedral ligand field are symmetry forbidden. Transferred to the MnV-WOC, this means that strongly localized Mn $d \to d$ transitions will be forbidden, whereas linear combinations of local transitions can acquire oscillator strength. This can be seen in the X_1 transition, which is a totally symmetric linear combination of Mn $d \to d$ transitions and thus is the brightest state. Above the low-energy region due to the Mn $d \to d$ transitions, all computed states can be described as either local excitations on the vanadate or charge transfer excitations involving the vanadate.

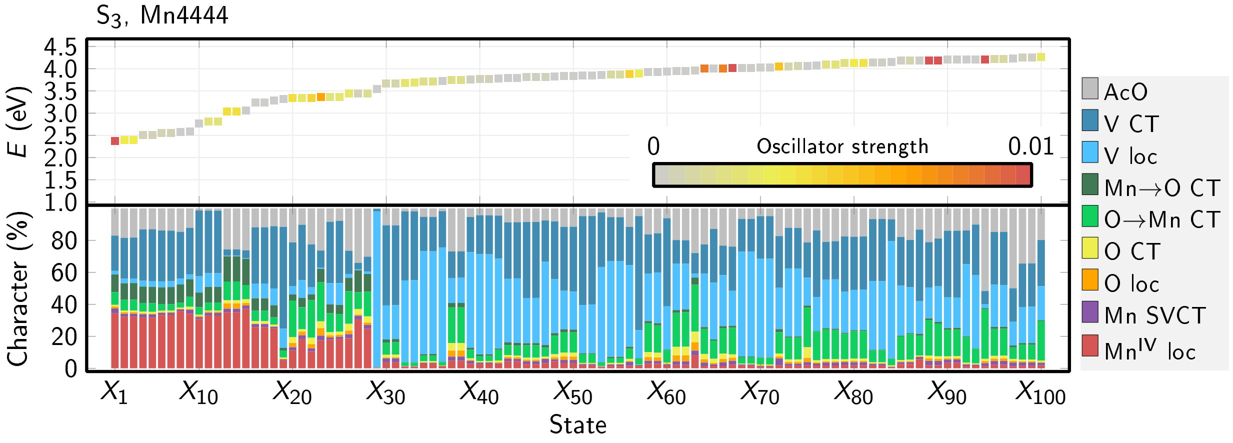

Figure S9: Excited-state and charge transfer analysis plot for the first 100 excited states of S_3 (Mn4444). The upper panel shows the excitation energies (vertical axis) and oscillator strength (color coded) for all states. For example, the first excited state ("X_1") has an energy of 2.38 eV and an oscillator strength of 0.016. The lower panel shows the charge transfer characters of all states. For example, the X_1 can be described as 34% local excitations on Mn^{IV}, 11% Mn→O charge transfer, 8% O→Mn charge transfer, 22% charge transfer to/from the vanadate moiety, and 17% excitations involving the acetates. Hence, the X_1 can be classified as a Mn^{IV} local excitation (the other contributions are due to the significant orbital delocalization).

In Figure S10, we show an analogous plot for the Mn3444 oxidation state. Here, the most interesting aspect is the presence of four local $d \to d$ transitions on the Mn^{III} atom (dark red). These four transitions arise because Mn^{III} has four occupied and one virtual d orbital. Most notably, the first excited state is such a transition and has an excitation energy of only 1.7 eV (730 nm), much lower than all other states. This transition is due to the excitation of the extra electron from the d_{z^2} orbital (along the Jahn–Teller axis) to the $d_{x^2-y^2}$ orbital (perpendicular to the Jahn–Teller axis). Its excitation energy provides a reasonable measure for the extent of the Jahn–Teller splitting of the two orbitals—the higher the excitation energy, the stronger is the splitting of the orbitals and the stronger the ligand field on that Mn atom is. However, as this low-energy

state does not mix with other transitions, it is very much localized on the Mn^{III} atom and hence is a rather weak (although not completely forbidden) transition by virtue of the LaPorte rule. It is also worth mentioning that no states with predominent intra-valence charge transfer (IVCT) character were found in our calculation, although these are often discussed for mixed-valence poly-nuclear metal complexes.

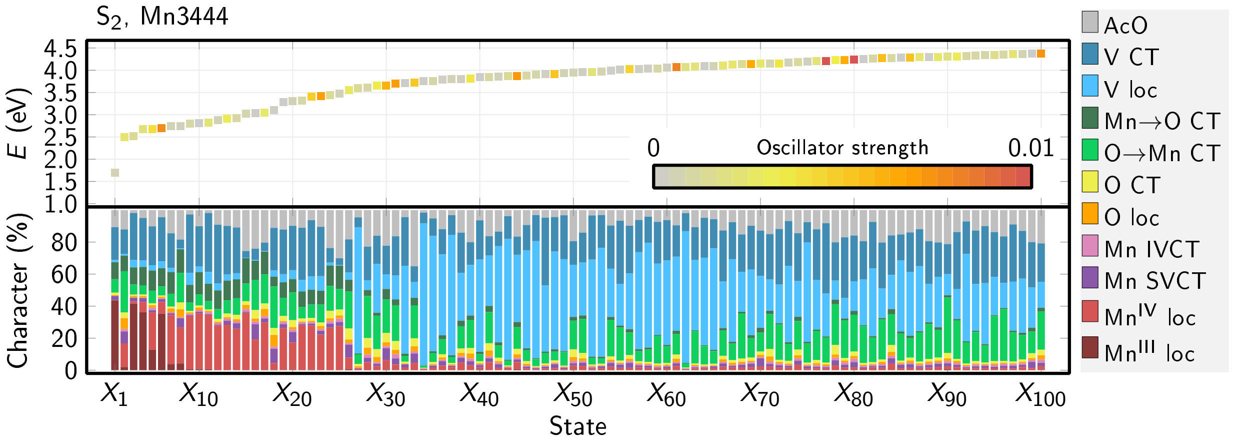

Figure S10: Excited-state and charge transfer analysis plot for the first 100 excited states of S_2 (Mn3444).

For the lower oxidation states Mn3344 to Mn3333 (Figures S11 to S13), the same trends can be observed. For each Mn^{III} atom, there is an additional low-energy excitation present, with an energy of about 1.5–1.7 eV (730–830 nm) for non-apical Mn atoms and 1.1–1.3 eV (950–1130 nm) for the apical Mn atom. This significant difference is due to the fact that the apical Mn atom is coordinated by the three acetate ligands, which are weaker ligands than the vanadate, and therefore for the apical Mn atom the splitting between d_{z^2} and $d_{x^2-y^2}$ orbitals is smaller. For all oxidation states, the excitations above 3.5 eV (350 nm) are dominated by transitions involving the vanadate. Thus, the UV part of the spectrum of MnV-WOC can be expected to be consistent with other UV/Vis spectra recorded for oligovanadates.

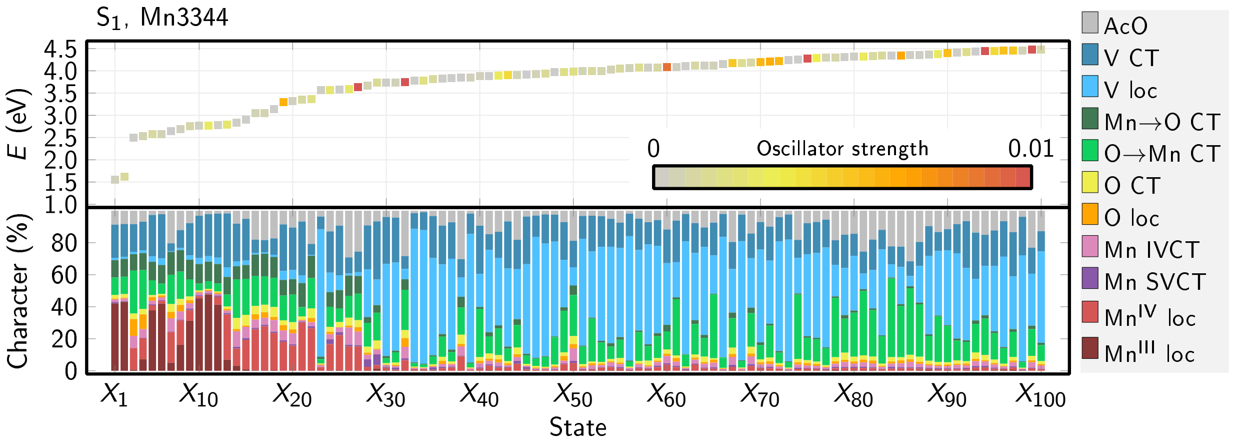

Figure S11: Excited-state and charge transfer analysis plot for the first 100 excited states of S_1 (Mn3344).

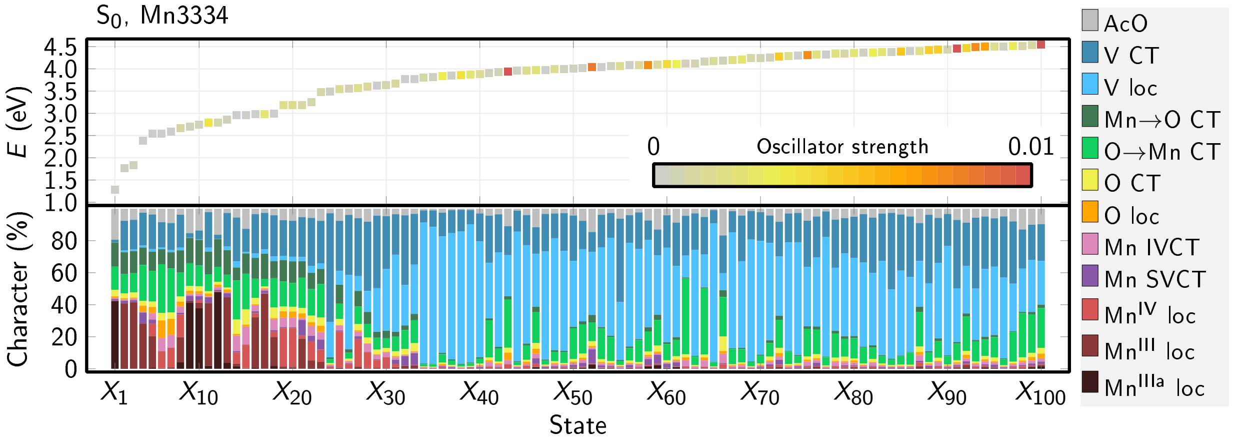

Figure S12: Excited-state and charge transfer analysis plot for the first 100 excited states of S_0 (Mn3334).

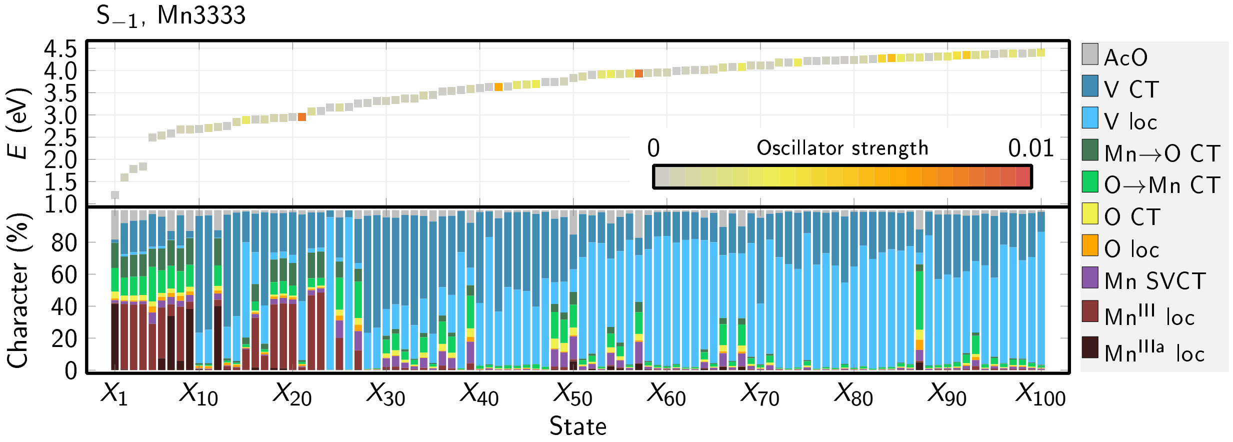

Figure S13: Excited-state and charge transfer analysis plot for the first 100 excited states of S_{-1} (Mn3333).

To illustrate the above discussion about the excited states, in Figure S14 we present the most contributing natural transition orbitals for the first excited state in the Mn4444 oxidation state and the first, third, and fifth excited state in Mn3444. For Mn4444 (Figure S14a), the excitation is primarily a transition from a linear combination of occupied Mn d orbitals (plus a p orbital on the apical oxygen) to unoccupied Mn d orbitals. One can see that this transition involves delocalized orbitals involving all symmetry-equivalent Mn atoms.

The first excited state in panel (b) shows a localized $d \to d$ transition on the Mn^{III} atom, involving the two orbitals that became non-degenerate through the Jahn–Teller effect. As the orbital energies of Mn^{III} are rather different from Mn^{IV}, there is no mixing of d orbitals of different atoms and hence the transition is strongly localized. The X_3 transition in panel (c) is another $d \to d$ transition on the Mn^{III} atom, again localized. The X_5 transition in panel (d) on the contrary is a transition that is delocalized over the remaining non-apical Mn^{IV} atoms, qualitatively similar to the X_1 transition in Mn4444 (a).

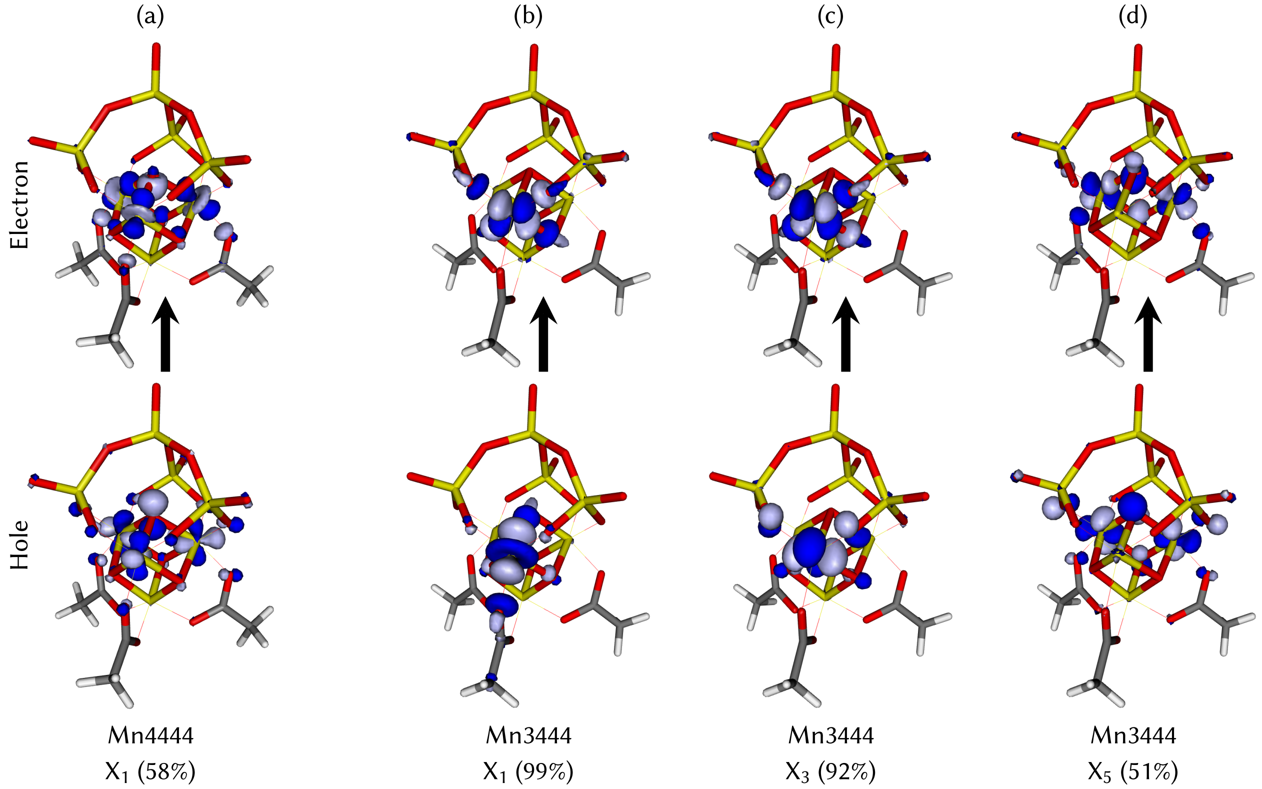

Figure S14: Depiction of natural transition orbitals for selected transitions in Mn4444 and Mn3444. The bottom orbitals are the hole orbitals (from where excitation takes place), the top orbitals are the electron orbitals (where excitation goes to). The labels give the oxidation state, excited-state numbering, and contribution of the shown NTO pair to the transition.

S2.4 Experimental UV/Vis spectrum

For comparison purpose, here we show the UV/Vis spectrum of the pristine precatalyst as reported by Schwarz et al.[1] (for technical details, please refer to the supporting information of Ref. 1). The spectrum is reproduced in Figure S15. It shows a very weak band at about 650–850 nm, a shoulder at 484 nm, and a much more intense shoulder at about 268 nm.

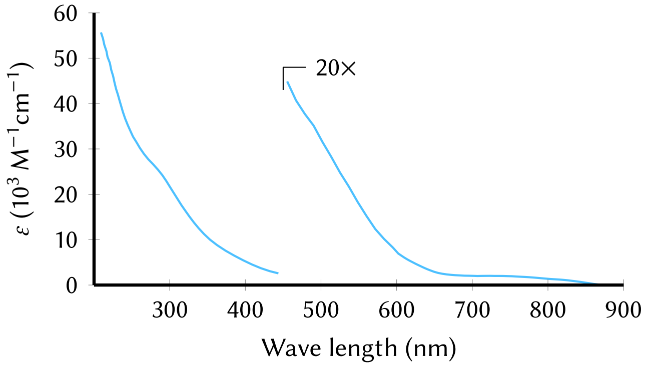

Figure S15: UV/Vis spectrum of the pristine precatalyst (Mn3344) in acetonitrile solution, data obtained from the supplementary information of Ref. 1. λ_{max1}=268 nm, ε=28900 $M^{-1}cm^{-1}$ (shoulder); λ_{max2}=484 nm, ε=2250 $M^{-1}cm^{-1}$ (shoulder); λ_{max3}=778 nm, ε=155 $M^{-1}cm^{-1}$.

Supplementary References

[1] B. Schwarz, J. Forster, M. K. Goetz, D. Yücel, C. Berger, T. Jacob, C. Streb, *Angew. Chem. Int. Ed.* **2016**, *55*, 6329–6333.
